# Composite Hydrogels with Embedded Electrospun Fibers as Drug Delivery Systems

**DOI:** 10.3390/gels11100826

**Published:** 2025-10-14

**Authors:** Paul Codrin Fuioaga, Delia Mihaela Rata, Tabinda Riaz, Guadalupe Rivero, Gustavo A. Abraham, Leonard Ionut Atanase

**Affiliations:** 1 “Cristofor Simionescu” Faculty of Chemical Engineering and Environmental Protection, “Gheorghe Asachi” Technical University of Iasi, 700050 Iasi, Romania; codrin_paul@ymail.com; 2 Faculty of Medicine, “Apollonia” University of Iasi, 700511 Iasi, Romania; delia.rata@univapollonia.ro; 3 Institute of Polymer and Textile Engineering, University of the Punjab, Lahore 54590, Pakistan; tabindaatif.ipte@pu.edu.pk; 4 Research Institute of Materials Science and Technology, INTEMA (UNMdP-CONICET), Av. Colón 10850, Mar del Plata B7606BWV, Argentina; grivero@fi.mdp.edu.ar (G.R.); gabraham@fi.mdp.edu.ar (G.A.A.); 5 Academy of Romanian Scientists, 050045 Bucharest, Romania

**Keywords:** electrospinning, hydrogels, composites, multilayer, 3D printing

## Abstract

Hydrogel/electrospun polymer nanofiber composites (HENC) integrate the advantages of both components. Hydrogels provide high water content, biocompatibility, and tunable drug release, while electrospun nanofibers offer a high surface area, loading capacity, customizable morphology, and opportunities for functionalization. Nanofibers can also be incorporated into hydrogels as 3D-printable inks. Together, these features create biomimetic composites that modulate drug release and mimic native tissues. This article reviews electrospinning fundamentals, limitations, preparation methods for HENC, and their applications in drug delivery, as well as future perspectives for developing advanced functional materials with improved therapeutic efficacy, controlled release kinetics, and broad biomedical adaptability.

## 1. Introduction

In recent years, the convergence of hydrogel and fibrous materials has opened new frontiers in the development of advanced drug delivery systems [1]. Fiber-based materials are critically important in biomedical applications due to their high surface-area-to-volume ratio, tunable porosity, and ability to mimic the extracellular matrix. These features promote cell adhesion, proliferation, and differentiation, making them ideal for tissue engineering scaffolds. Additionally, their structural versatility allows for controlled drug loading and release, enabling targeted and sustained delivery of therapeutic agents. Among the nanofiber production techniques, electrospinning (ES) is the most convenient technique for producing hydrogel/polymer nanofiber composites due to its versatility and precise control over fiber morphology, as well as the incorporation of a wide range of polymers and hydrogels, and the encapsulation of drugs or bioactive molecules for controlled release.

ES can produce ultrafine fibers from polymer solutions or melts by applying a high-voltage electric field [2]. Both natural and synthetic polymers can be electrospun, and drugs can be loaded either by blending them into the polymer solution before spinning (monolithic loading) or by surface functionalization post-spinning [2]. Electrospun fibers offer high surface area-to-volume ratios, customizable architectures, and the ability to encapsulate a wide range of bioactive compounds. When combined with the unique properties of hydrogels, this convergence has opened new frontiers in the development of advanced drug delivery systems [3,4].

Hydrogels are three-dimensional, hydrophilic polymer networks capable of absorbing significant amounts of water or biological fluids until reaching a swelling equilibrium determined by the balance between osmotic forces and the polymer network’s elasticity [5]. They can be derived from natural polymers (e.g., gelatin, alginate, chitosan, hyaluronic acid), semi-synthetic polymers (e.g., gelatin methacrylate [GelMA], hydroxypropyl methylcellulose [HPMC]), or synthetic ones (e.g., polyethylene glycol [PEG], polyvinyl alcohol [PVA], polyacrylamide) [6]. Semi-synthetic polymers combine the inherent bioactivity and biocompatibility of natural polymers with the tunable mechanical and chemical properties of synthetic polymers, making them especially versatile for biomedical applications. Crosslinking, either physical (ionic, hydrogen bonding) or chemical (chemical reaction to form covalent bonds, photochemical, enzymatic), is used to form the network structure. The degree and type of crosslinking directly influence the hydrogel’s mechanical strength, degradation rate, and drug release behavior [7]. Hydrogels, with their high-water content, biocompatibility, and tunable physical properties, have long been valued in biomedical applications. However, their relatively weak mechanical strength and uncontrolled drug release profiles have posed limitations for certain therapeutic uses [8]. To address these challenges, the incorporation of electrospun fibers into hydrogel matrices has emerged as a promising strategy to engineer composite systems that synergize the advantages of both materials. When embedded within hydrogels, these fibers can reinforce the structural integrity of the matrix and enable controlled, localized drug delivery through diffusion or degradation-mediated mechanisms. Such composite systems can be precisely engineered to respond to environmental cues, such as pH, temperature, redox reaction, light, or enzymatic activity, further enhancing their potential for targeted and sustained therapeutic release [9].

The integration of electrospun fibers into hydrogel matrices represents a cutting-edge strategy in the design of multifunctional drug delivery systems. Each component contributes distinct and complementary properties: hydrogels provide a biocompatible, hydrated environment favorable for cell interaction and sustained drug release, while electrospun fibers add mechanical strength, provide structural control (e.g., porosity, fiber alignment), modulate drug release kinetics (e.g., reduce burst release) and allow targeted drug capabilities (e.g., by spatial localization, surface functionalization, encapsulation of multiple drugs) [10].

The fabrication of hydrogel/electrospun polymer nanofiber composites (HENC) involves embedding the electrospun fibers within a hydrogel matrix using different strategies [1,11]: (i) in situ encapsulation: electrospun fibers are collected and directly embedded into the hydrogel precursor solution before crosslinking. Upon gelation, the fibers are immobilized within the matrix; (ii) layer-by-layer assembly: alternating layers of hydrogel and electrospun fiber mats are stacked to form a multilayered composite, offering spatial control over drug distribution; (iii) coaxial systems: in more advanced designs, coaxial electrospinning can produce core–shell fibers, where the core contains the drug and the shell modulates its release. The shell can also incorporate hydrogel properties to further control drug delivery.

**Figure 1:**
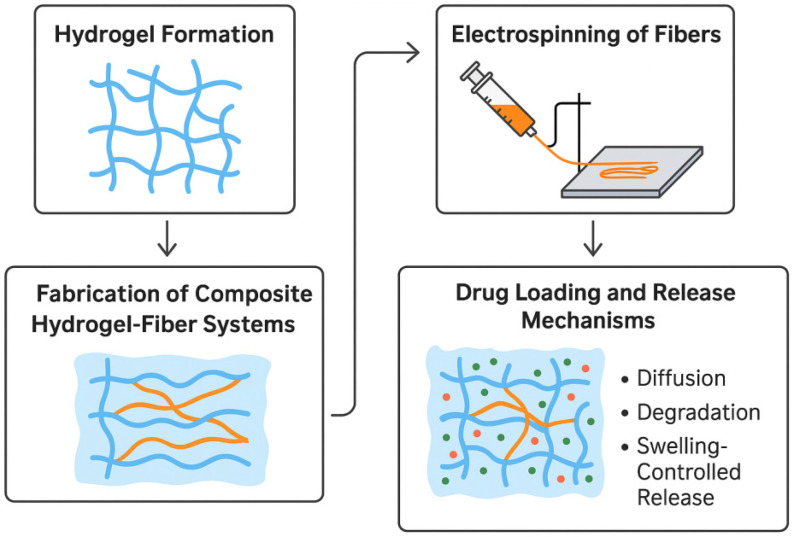
Process diagram composite hydrogels with embedded electrospun fibers preparation for drug delivery.

The embedding process must ensure homogeneous distribution and strong interfacial bonding between fibers and the hydrogel to maintain mechanical integrity and control release profiles, as illustrated in Figure 1.

This review focuses on the design, fabrication, and characterization of composite hydrogels embedded with electrospun fibers as novel drug delivery platforms. By discussing their physicochemical properties, drug release kinetics, and biocompatibility, it was possible to demonstrate the potential of these multifunctional systems in addressing the limitations of conventional drug carriers and advancing the field of personalized medicine.

## 2. Electrospinning Technique

### 2.1. Overview

ES is an electrohydrodynamic (EHD) process that has become an efficient and promising method to produce one-dimensional nanomaterials in the form of nanofibers for biomedical and nanotechnology-related applications [12,13,14,15]. Research areas that have greatly benefited from this technique include tissue engineering [16,17,18,19,20,21], wound healing [22,23,24], drug delivery platforms (involving cells, gene, nucleic acids, growth factors and drugs) [25,26,27], biosensors [14,28,29], bacteria immobilization [30], enzyme encapsulation [31] and many other explored applications [32,33]. Recently, Sánchez Cerviño reported on the use of EHD techniques both for the immobilization and for the synthesis of vesicles in a non-conventional manner [34].

The extensive application of ES is attributed to the enormous versatility of the process, which is capable of producing polymer-based nanofibers (from natural or synthetic polymers, polymer blends or nanocomposite materials), as well as ceramic, metal, carbon-based and semiconducting materials. Electrospun nanofibers exhibit a unique combination of properties, including an extremely high surface area-to-volume ratio, a complex porous structure with tunable porosity and full pore interconnectivity, and controllable topography and morphology at both nano- and microscales, which contribute to their wide-ranging applications [35]. By carefully adjusting operating conditions, setup, and the composition of the polymer solutions, ES allows the formation of various structures, such as core–shell, side-by-side, multilayer, and fiber assemblies [36]. Additionally, the development of surface-functionalized scaffolds and 3D nanofibrous structures remains among the most active research areas in the field, as these materials offer enhanced cellular interactions, controlled mechanical properties, and tailored microenvironments, which are critical for applications in tissue engineering and drug delivery systems [37,38].

Upscaling the production of electrospun nanofibers presents both significant opportunities and challenges, especially considering their wide range of applications in areas such as filtration, tissue engineering, drug delivery, and protective textiles. Several strategies have been explored to overcome the limitations of traditional lab-scale ES techniques, which are often limited by low production rates [39,40].

### 2.2. ES Fundamentals

Briefly, ES is based on the application of a strong electric field to a viscoelastic fluid, typically a polymer-based solution or polymer melts. When a critical voltage is reached, the electrostatic force overcomes the surface tension of the charged fluid, creating a conical tip (Taylor cone). This results in the ejection of a microjet of fluid that is accelerated towards a collector, which is either grounded or has an opposite polarity. In solution electrospinning, as the charged jet travels towards the collector, solvent evaporates. Meanwhile, the whipping (bending) instability, induced by the uneven distribution of surface charges, causes rapid stretching and elongation of the jet, contributing to fiber thinning and ultimately leaving behind solid ultrafine fibers [41].

Depending on the specific conditions of the polymer fluid (solution, emulsion, suspension, or melt) and the processing parameters, the microjet can either form solid micro-/nanofibers or micro-/nanoparticles. At low concentrations (below a critical concentration specific to each polymer solution), polymer entanglements are absent, and the jet can break up into fine droplets, resulting in the formation of particles through a process known as electrohydrodynamic atomization or electrospraying [42,43]. ES setups are highly versatile and vary greatly depending on the specific application. The basic configuration consists of five essential components: a fluid reservoir (typically a syringe containing the polymer solution or melt), a single nozzle through which the fluid is extruded, a syringe pump that controls the flow rate of the solution or melt at a constant, precise rate, a high-voltage power supply to create the required electric field, and a collector, a grounded or oppositely charged flat surface positioned at a specific distance from the nozzle [43]. More advanced setups may include additional components, such as multiple nozzles (for co-electrospinning or multi-material fibers), rotating collectors (to produce aligned or continuous fibers), and environmental controls (to regulate humidity and temperature, which affect fiber quality). These modifications enhance the versatility of electrospinning, allowing the production of complex fiber architectures.

The ES process is governed by a complex set of interrelated operational parameters, which can be broadly classified into three categories: intrinsic (specifically solution properties), processing, and environmental parameters [44,45].

Solution properties play a key role in determining the quality and characteristics of the resulting fibers. These properties include the composition of the polymer/blends and solvent/solvent mixture, the viscosity (which is primarily influenced by the polymer concentration and molecular weight), electrical conductivity (which depends on the nature of the polymer and any dissolved conductive additives), surface tension, and the boiling point of the solvent. Viscosity and conductivity control the fluid’s ability to be drawn into fine fibers, while surface tension affects the stability of the jet, and the solvent boiling point influences the evaporation rate, all of which contribute to the final fiber morphology [46,47]. Furthermore, the presence of dispersed polymer, ceramic, or metallic nanoparticles, soluble drugs or biomacromolecules, emulsified phases, or other components introduces additional complexity to the intrinsic properties of the electrospun fluid.

Processing parameters are equally important and have a significant influence on fiber formation. In the simplest case, the most relevant operating parameters include applied voltage, the distance between the charged nozzle and the collector, and the flow rate of the polymer solution. For example, increasing the voltage typically increases the stretching of the jet, leading to thinner fibers, while the nozzle-to-target distance determines the time available for the jet to stretch and for the solvent to evaporate before the fibers are collected. In far-field electrospinning, the collector is positioned at a relatively large distance from the needle, allowing the charged polymer jet to undergo extensive stretching and bending instabilities, which produce randomly oriented nanofibers. In contrast, near-field electrospinning involves a much shorter needle-to-collector distance, reducing jet instabilities and enabling precise deposition of fibers with controlled alignment and patterning. The flow rate, if too high, can lead to beads or thick fibers due to insufficient stretching or solvent evaporation; if it is too low, it can result in inconsistent fiber production.

Collector type and shape, multi-nozzle arrays, or nozzleless systems are examples of the versatility in EHD techniques, allowing for greater customization in fiber production. The collector can be designed in various forms, such as flat, cylindrical, or rotating setups, each influencing the fiber alignment, texture, and structure. Multi-nozzle arrays enable the simultaneous extrusion of multiple polymer solutions, which can enhance production efficiency and allow for the fabrication of complex, multi-layered, or blended fibers. Nozzleless EHD processes, on the other hand, simplify the setup by eliminating the need for individual nozzles. This design enhances scalability for certain polymers, reduces the risk of clogging issues commonly encountered with multi-nozzle systems, and allows for a more continuous and efficient production process, particularly beneficial in high-throughput applications.

Finally, environmental parameters, particularly temperature and relative humidity, have a significant impact on the ES process. The proper value of these parameters can vary depending on the chemical structure of the polymer or solvent used. For instance, temperature influences the viscosity of the solution and the rate of solvent evaporation, both of which affect the fiber diameter and morphology. Higher temperatures generally lower the viscosity and accelerate evaporation, leading to thinner fibers. Similarly, relative humidity plays a crucial role by affecting the evaporation rate of the solvent. High humidity levels can lead to incomplete solvent evaporation, resulting in wet or fused fibers, while low humidity accelerates evaporation, potentially leading to brittle fibers or even jet instability. The interplay of these environmental factors must be carefully controlled, particularly in the case of polymers sensitive to humidity or temperature fluctuations, to tailor fiber properties effectively [46,47].

### 2.3. Incorporation of Drug into Nanofibers

Nanofibers present a highly attractive platform for developing advanced drug delivery systems tailored to specific therapeutic needs. These systems can encapsulate and release a broad range of bioactive agents and biomacromolecules, including peptides, proteins, genes, and other biomacromolecules, facilitating targeted and sustained release profiles [48,49]. Drug-loaded electrospun nanofibers can be obtained by different techniques, each offering distinct advantages and limitations in terms of encapsulation efficiency, release kinetics, and stability (Table 1). Drug release behavior is affected by both polymer and drug-related factors.

The ES of drug/polymer blends is the simplest method to incorporate an active bioagent within submicrometric fibers. The chosen solvent should dissolve both the polymer and the agent without affecting its functionality through degradation, denaturing, etc. The drug is directly incorporated into the pristine fluid and then electrospun. When the solvent evaporates, the agent remains inside the polymer matrix. However, the relative physicochemical features, solubility, and interactions among them will determine the extent of homogeneity of the dispersion in the final fibers. The compatibility and drug content should be adjusted to avoid phase separation and burst release effects.

Core–sheath fibers are excellent options for incorporating certain drugs or agents into incompatible polymer-based fibers. The inherent phase separation provides additional protective barriers that usually extend the release profiles. Emulsion ES uses a stable emulsion as a fluid. Normally, additional components, such as surfactants, should be added to assure the kinetic stability of the emulsion, so that the size of the droplets of the discrete phase (containing the drug) does not change in time during processing. As tension is applied, the solvent evaporates while the droplets stretch, and drug-rich domains coalesce to form a core [31,52,53,54].

Coaxial ES [55,58] requires a concentric nozzle so that two different fluids are simultaneously infused, leading to core–sheath fibers after electrospinning. Drugs with different chemical natures could be eventually incorporated into a single core–sheath material, given that the contact between the two phases is minimized. However, besides the compositional optimization of both phases, the processing parameters should be precisely tuned. In particular, the relative flow rate would determine the thickness ratio, thus affecting the drug release profiles.

Post-processing modifications can be made to fibers in order to incorporate certain agents into the surface using physical or chemical methods. The interconnected porosity of the nanofibrous membranes allows for coatings, adsorption, chemical reactions, and/or crosslinking on the fibers [56,57]. In all cases, the protocols should be thoroughly reviewed and, if necessary, updated to guarantee that the drug retains its intended functionality, stability, and efficacy throughout every stage of the process—from initial preparation and handling to final application or administration.

Finally, the drug of interest can be previously entrapped in supramolecular structures, such as nanoparticles, nanogels, or complexes, which are then further incorporated into a polymeric fluid for electrospinning [27]. This pre-confinement strategy increases drug protection and stability. Furthermore, the versatility of these approaches may be used to provide the delivery systems with components with selective sensitivity to given external stimuli (pH, heat, humidity, magnetic stimulus), with the aim of precisely triggering the release.

### 2.4. Emerging Challenges in ES

Despite extensive research on electrospun scaffolds, restricted pore interconnectivity and limited 3D organization can hinder their effectiveness in supporting the regeneration of thick, complex tissues. In response, innovative strategies are being proposed to enhance scaffold performance (Table 2). Bongiovanni et al. [38] reviewed the combination of ES with other fabrication techniques to produce 3D polymeric/composite nanofibrous scaffolds with micro/nanotopographical features to improve mechanical strength and biological performance. These challenges arise from issues related to the mechanical strength and cellular infiltration capacity of nanofiber-based scaffolds, which are often insufficient for fully mimicking the structure and function of native tissue. This combined approach aims not only to strengthen the mechanical properties but also to enhance cell attachment, proliferation, and differentiation, which are crucial for the successful integration of engineered tissue with the host.

Various research groups have investigated the creation of complex 3D structures using 3D printing and ES in a synergistic approach [59] and cell ES and 3D printing combination [60]. Dalton et al. [61] reviewed the development of hybrid fabrication techniques toward hierarchical tissue constructs, highlighting the importance of the combination of fabrication technologies to complement each other.

In addition, green ES constitutes another big challenge in the design of advanced manufacturing drug delivery systems. This involves the use of benign or non-toxic solvents instead of typical toxic organic solvents. Using molten polymers for ES is a solvent-free strategy, but it is limited to certain polymers. Melt ES writing (MEW) is a hybrid technology that combines ES and microextrusion, enabling fiber diameters ranging from 800 nm to 150 μm.

Stimuli-responsive nanofibers are an emerging class of materials that can undergo physical or chemical changes in response to external triggers such as pH, temperature, light, or magnetic fields. By integrating these responsive polymers into nanofibrous structures, it is possible to create scaffolds or delivery systems that adapt dynamically to their environment. Such fibers are particularly valuable in biomedical applications, enabling on-demand drug release, controlled swelling, or modulation of cellular behavior, thereby enhancing the functionality of tissue engineering constructs and therapeutic platforms [62,63,64]. In addition, ES nanofibers can combine biomechanical and topographical stimuli, incorporating proper chemical/biological signals by surface modification. In this sense, fiber orientation (random and oriented fibers), fiber size, and topography can drive cell growth from a spatial standpoint [65].

**Table 2:** Recent literature on the incorporation of bioactive agent-loaded electrospun nanofibers and their applications.

System Processing	Bioactive Agent	Polymer	Aim	Main Focus	Application Area	Ref.
Uniaxial/coaxial electrospinning	hydrocortisone	Polycaprolactone (PCL)	Alternative to cream-based therapies for 24 h release	In vitro release, ex vivo penetration, and permeation on porcine skin	Skin disease therapy	[66]
Twin-screw melt granulation of loaded-electrospun fibers	itraconazole	poly-vinylpyrrolidone (PVP)-vinyl acetate, hydroxypropyl methylcellulose (HPMC)	To improve the dissolution of poorly water-soluble drugs	Post-processing, stability	Biopharmaceutics industrial processing	[67]
Uniaxial electrospinning	pregabalin	PVP, HPMC, polyvinyl alcohol (PVA)	Drug release and solubility control	Release from water-soluble polymers	Drug delivery systems	[68]
Uniaxial electrospinning	budesonide	PCL, poly(D,L-lactide-co-glycolide) (PLGA)	Morphology control, fiber diameter prediction	Processing parameters, release	Drug delivery systems	[69]
Nanoparticles in nano/microfibers	Gentamicin, dexamethasone	Poly(lactic acid) (PLA) fibers embedded with halloysite nanotubes	Dual hydrophilic/hydrophobic release	Drug release	Drug delivery systems	[70]
Uniaxial/coaxial electrospinning	captopril	Ethyl cellulose (EC)	Up-scaling	Bench-top and scale-up method, release	Drug delivery systems	[71]
Uniaxial electrospinning as coating	vancomycin	PVA	Antibacterial implant coating	Antibacterial properties	Biomaterial devices	[72]
Uniaxial electrospinning (flat/drum collectors)	melatonin	PCL	Fabrication optimization for up-scaling	Formulations	Wound healing	[73]
Microsphere suspension electrospinning	ampicilin, rhodamine	PVP	To increase the load capacity, to eliminate the burst effect	Manufacturing method	Drug delivery	[74]
Mono-, bi-, and tri-layer fibers	Acetaminophen	Cellulose acetate (CA)	Gradient drug distribution	Release profiles	Drug delivery	[75]
Uniaxial electrospinning	sulfamethoxazole	PVP, PVA, HPMC	Drug encapsulation	Release profiles, solubility	Drug delivery	[76]
Uniaxial, coaxial and layer-by-layer electrospinning	tofacitinib	PCL	3 days- release	Morphology, release, permeation	Skin disease therapy	[77]
High-speed electrospinning	doxycycline-hyclate	2-hydroxypropyl-β-cyclodextrin (HP-β-CD)	Quality assurance system, upscaling	Load, morphology, monitoring methods	Drug delivery	[78]
Electrospun fiber-in/on -film composites	phenytoin	Ethyl cellulose	Tailorable in vitro drug release	Release profiles	Wound healing	[79]
Film based on electrospun fibers	quercetin	Eudragit EPO/sodium hyaluronate	Antioxidant and antiperoxidation strategies	Antioxidant properties	Drug encapsulation	[80]
Core–shell electrospun nanofibers coated with silver nanoparticles	rifampicin	PCL	Antibacterial effect	Morphology, antibacterial properties	Tissue engineering	[81]

## 3. Composite Materials Based on ES Nanofibrous Mats and Hydrogels

### Platforms Combining Hydrogel 3D Printing and Electrospinning

**Figure 2:**
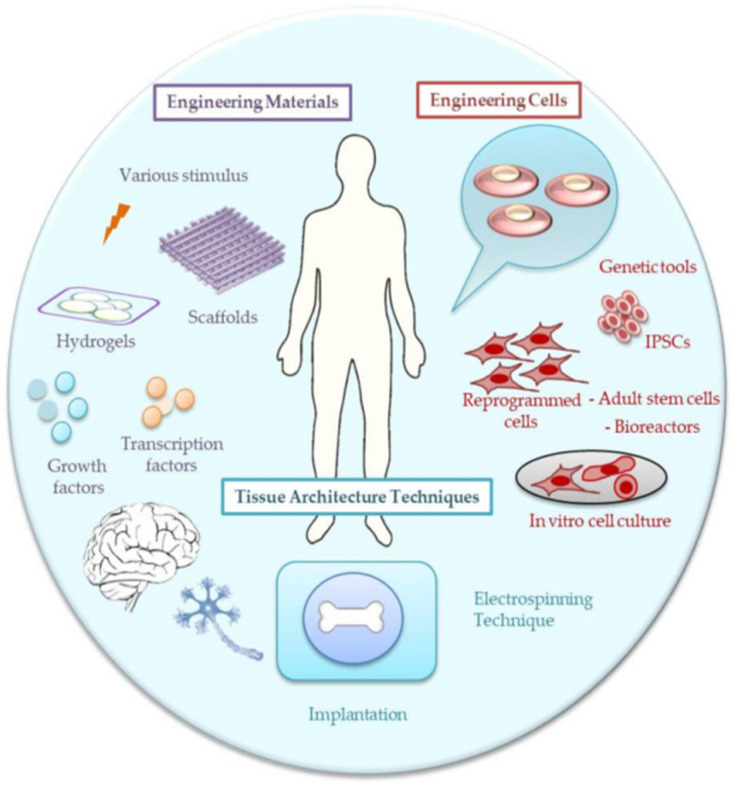
Incorporation of cell cultures into the electrospun composite hydrogels for tissue engineering applications [85].

The world is facing an ever-growing demand for smart solutions to biomedical challenges that pave the way for the design and fabrication of novel and multifunctional products that have synergistic performance and an expanded scope of applications. Composite hydrogels are one of these innovations that combine multiple components to provide versatility in terms of mechanical strength, swelling properties, and biological functionality. These hybrid materials integrate hydrophilic polymers with bioactive additives or nanostructures, providing customizable properties for various biomedical and industrial applications. Their ability to mimic biological tissues, support drug delivery, and enable tissue regeneration has positioned composite hydrogels as essential tools in healthcare, environmental science, and regenerative medicine. Composite hydrogels are extensively utilized in tissue engineering, wound healing, and drug delivery systems. For instance, the tragacanth gum (TG) hydrogel incorporating silver sulfadiazine (SSD) and Aloe Vera (AV), combined with electrospun PAN nanofibers, resulted in enhanced therapeutic and hydrophilic characteristics. This sandwiched wound dressing showed good mechanical behavior and cell viability with no signs of cytotoxicity, making it ideal for biomedical applications. Similarly, photocrosslinked chitosan hydrogels combined with curcumin and soy protein isolate (SPI) nanofibers demonstrated anti-inflammatory effects and structural support, facilitated epidermal formation, and increased collagen density, thus accelerating wound healing [82,83,84]. For tissue engineering applications, optimal biochemical properties, such as biocompatibility, biodegradation, and mechanical properties related to porosity, stability, and morphology, are required to facilitate both cell infiltration and cell-material interaction (Figure 2).

Electrospun nanofibers can be transformed into hydrogels by categorizing them on the basis of the types of polymers used; also, electrospun nanofibers can be joined with hydrogels to form composite structures to perform versatile functions in biomedical applications. In fact, hydrogels fortified with nanoparticles are promising candidates for sustained drug release and tissue regeneration. In a recent study, methacrylate gelatin hydrogels combined with poly-(lactic acid co-trimethylene carbonate) nanofibers loaded with Epincidine-1@chitosan nanoparticles acted as temperature-responsive, self-contracting composite hydrogel dressing. This system showed cytocompatibility, antioxidant, anti-inflammatory, and antibacterial properties, thereby promoting collagen deposition and supporting rapid healing [86]. A hydrogel film of methacrylated gelatin (GelMA) loaded with silver nanoparticles (AgNPs) poly(vinylpyrrolidone) coated iridium nanoparticles (PVP-Ir-NPs) was electrospun in an aligned manner and photo-crosslinked afterwards. The prepared film possessed high water content and biocompatibility, demonstrating cell adhesion and proliferation. Moreover, the incorporated AgNPs and nanozymes, PVP-Ir-NPs, acted as catalytic entities for various kinds of reactive oxygen species (ROS) and showed antibacterial activity [87]. For bone tissue regeneration and to impart osteoinductive properties, a novel nanocomposite scaffold was prepared by sandwiching PLGA/PEG nanofibers between two gelatin-based hydrogel layers with tantalum nanoparticles. The surface roughness induced by tantalum NPs helped in cell adhesion and migration, and cells expressed the typical markers of differentiated osteoblasts, with production of osteocalcin and deposition of collagen for the formation of ECM [88].

**Figure 3:**
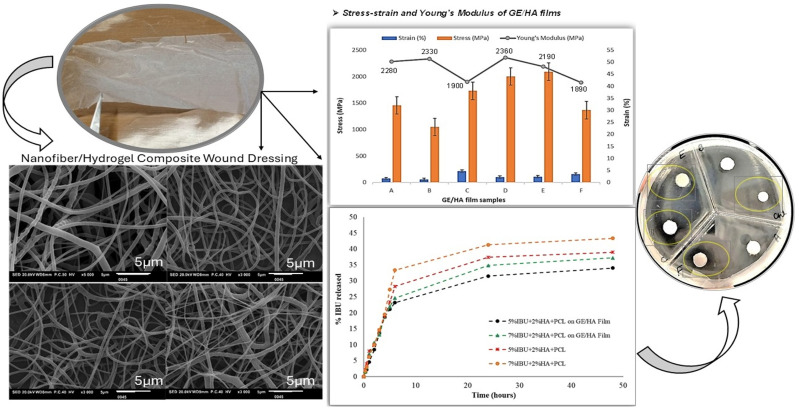
Characterization results (SEM images, tensile testing, drug release profile, and antibacterial assay against *E. coli* where zones of inhibition (ZOI) are highlighted by yellow circles) of bilayer composite hydrogel with ibuprofen-loaded PCL nanofibers on Gel/HA hydrogel base using needleless NanoSpider electrospinning technique. Redrawn from reference [92].

The combination of electrospun nanofibers with hydrogels provides an augmented benefit of tailoring the resultant characteristics of the composite structure, such as mechanical strength and degradation properties. Nanofibers with 3D networks and fine morphology resemble the extracellular matrix, which supports cell proliferation and adhesion for tissue regeneration and wound healing [89,90]. The HA-valsartan hydrogel, integrated with PLA nanofibers, promotes fibroblast activity and re-epithelization, showcasing its potential in managing diabetic wounds through sustained drug delivery and tissue regeneration [91]. Another study reported the physical adhesion technique to create a hybrid bilayer wound dressing by electrospinning PCL nanofibers loaded with Ibuprofen onto a wet gelatin/hyaluronic acid hydrogel base. The bilayer composite material showed a sustained Ibuprofen release for 48 h and antibacterial activity against *E. coli* bacteria, showing its potential as a wound dressing for inflammatory wounds [92]. Figure 3 illustrates some of the most important results concerning ibuprofen-loaded PCL nanofibers in a Gel/HA hydrogel.

Fine-tuning of nanofibers and the use of appropriate polymer blends in hydrogels make it possible to achieve a prolonged release of drugs or bioagents for several weeks. This aspect of electrospun composite hydrogels has paramount significance in biomedical applications when sustained and targeted drug delivery is required. A study is documented where a laminated hydrogel/nanofiber composite scaffold was developed using an amalgamation of Alg:Alg-Sul hydrogel and PLGA nanoparticles carrying Keratogenin (KGN) incorporated PCL/Gel electrospun mats, which exhibited superior mechanical properties and a linear sustained release of KGN within 30 days [93].

Encapsulation and delivery of therapeutic drugs via nanofibers is an effective strategy for targeted recovery and wound management [94]. There are different methods of drug loading into the nanofibers’ matrix, such as direct incorporation into the polymer solution or impregnating the prepared nanofibrous mats into the drug solution. The drug is either entrapped inside the nanofibers network or bonded onto the surface through hydrogen bonding, hydrophobicity, or electrostatic interactions. The rate of drug release highly depends on the nanofiber morphology, diameter, porosity, drug loading methods, and electrospinning parameters (polymer concentration, solution feed rate, applied voltage, needle to collector distance). Similarly, hydrogels can also act as ideal drug carriers and diffusers employing their inherent characteristics like hydrophilicity and swelling ratio [95]. The release of loaded drugs is regulated through their degree of crosslinking, degradation rate, and ionic strength. Hydrogels are not only biocompatible and biodegradable but also responsive towards different stimuli, like temperature, pH, and ionic charge of their release media [96]. The internal structure of hydrogels is porous and has channels that provide storage sites for drugs/cytokines, safeguard them from degradation and the effect of the external environment, thus prolonging their release over a period of time [97,98]. When electrospun nanofibers and hydrogels are used as one unit for drug delivery, they have multifold benefits that have the flexibility of loading the drug either in nanofibers or into the hydrogel, depending on their desired release and functionality. Examples of such composite hydrogels are reported; electrospun PCL nanofibers in a gelatin methacrylic anhydride (GelMA) hydrogel, with encapsulated angiogenic drug deferoxamine, promoted rapid healing of chronic diabetic wounds and had a faster ability to achieve hemostasis [99]. Likewise, polyamide 6 nanofibers grafted with polyacrylamide create antimicrobial and controlled drug release properties, making them suitable for wound healing and pharmaceutical innovations [100]. Composite systems like Fiber-Rop/Gel-Clo hydrogels with thermosensitive nanofibers support sustained drug release, ideal for post-surgical pain management and regional anesthesia [101]. In another design, polyacrylamide hydrogels combined with cellulose acetate nanofibers improve ibuprofen delivery, reducing burst release and ensuring localized therapeutic efficacy [102]. Elastin-based PLLA composites further showcased responsiveness to inflammation, enabling precise drug release and supporting regenerative medicine [103].

Composite hydrogels demonstrate versatility through their integration of bioactive components and tailored mechanical properties (Table 3).

Hybrid hydrogels incorporating polymers and nanofibers mimic ECM structures, crucial for bone, cartilage, and neural regeneration applications where controlled biodegradation and cell differentiation are essential. Fiber-reinforced hydrogels, like those combining PCL fibers and chitosan hydrogels, form 3D networks that mimic natural skin, offering moisture retention, flexibility, and infection prevention, thereby enhancing tissue regeneration [104]. Injectable hydrogels formed via Schiff-base reactions exemplify tunable gelation, enhanced mechanical strength, and bioactivity, suitable for minimally invasive tissue engineering and cartilage regeneration applications [105]. Moreover, hybrid hydrogels synthesized from natural-synthetic blends possess interesting features like biological compatibility and promising physical–mechanical properties. The composite hydrogel made from silk fibroin/PVA nanofibers and sodium alginate/gum tragacanth hydrogel infused with cardamom oil provides antibacterial properties and facilitates cell attachment, making it ideal for skin tissue engineering and wound healing [106]. The inclusion of nanostructures in composite hydrogels has significantly expanded their functionality. Short nanofiber-loaded hydrogels for localized vaginal delivery of anti-HIV drugs demonstrate immediate EFZ release and sustained RIT delivery, addressing global health challenges like HIV prevention. Meanwhile, hydrogels integrated with nanoparticles and nanofibers provide enhanced strength, flexibility, and bioactivity, ensuring their relevance in addressing complex biomedical needs [107].

An innovative 3D hybrid hydrogel was prepared by utilizing photo-crosslinkable gelatin nanofibers embedded with MNPs containing cells, coupled with a methacrylate-gelatin and hyaluronic acid hydrogel. The alignment of MNP-loaded nanofibers in the hydrogel was controlled by applying an external magnetic field. This hybrid hydrogel served as a scaffold to promote dermal fibroblast and exhibited myofibroblastic differentiation [108]. Superparamagnetic iron oxide nanoparticles (SPIONs) were incorporated in synthetic nanofibers before their electrospinning to produce highly aligned nanofibers under the effect of an applied magnetic field. Nanofibers with SPIONs were introduced in a dextran vinyl sulfone bulk hydrogel and were aligned using a magnetic field strength. This hydrogel composite was employed to direct the migration of spheroids from multicellular to disconnected single cells and multicellular clusters [109].

**Figure 4:**
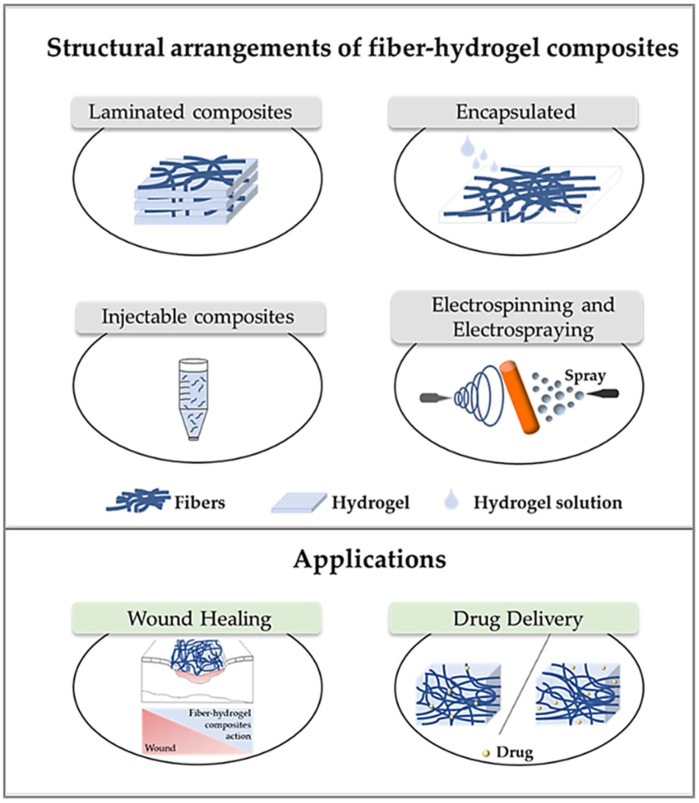
Fabrication techniques of electrospun composite hydrogels and their applications [129].

Different methods can be used for the preparation of composite hydrogels with embedded fibers, as shown in Figure 4.

The alignment of nanofibers can play a pivotal role in defining their final morphology and functionality during electrospinning. Nanofibers can be patterned in different ways to conform to their special application parameters. For instance, aligned PCL/Gelatin nanofibers, laminin-coated, were electrospun and embedded in hyaluronic acid hydrogel containing neural cell cultures for neural tissue engineering to restore the functionality of damaged neural tissue [130]. The anisotropic behavior of electrospun nanofibers can govern the alignment of cells, which can arrange themselves according to the morphology of nanofibers. This tunability of cells is highly desirable in certain tissue regeneration applications, like muscle and nerve cells. The length of the nanofibers dispersed in hydrogels can be controlled and kept short (usually in micrometers) to provide mechanical stability to the hydrogel and ensure permeability. Magnetic nanoparticles (MNPs) are used in biomedical products for their ability to interact with the applied magnetic field and can be incorporated in polymer solutions for their subsequent electrospinning into nanofibers. These MNPs are usually encapsulated with different cytokines.

**Figure 5:**
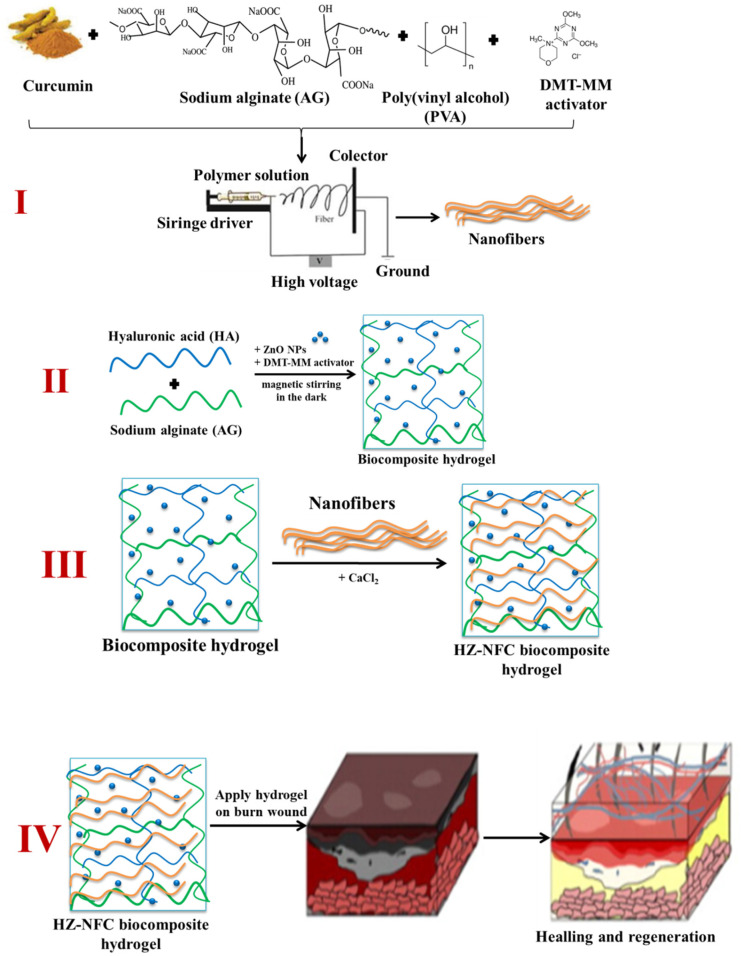
Schematic representation for the synthesis of biocomposite complex hydrogel with immobilized ZnO NPs and curcumin-loaded electrospun nanofibers.

Very recently, a new type of biocomposite complex hydrogel, based on hyaluronic acid and alginate, with immobilized ZnO NPs and curcumin-loaded electrospun nanofibers based on PVA and alginate was obtained by our team, as illustrated in Figure 5 [131].

These hydrogels are biodegradable, hemocompatible, non-cytotoxic, and non-irritating and have good antimicrobial activity, which makes them potential candidates for wound healing [131].

The remarkable impact of electrospun composite hydrogels in diverse biomedical applications is undeniable, yet they have certain limitations that restrict their performance in specific cases. Compromised mechanical properties, poor cell adhesion, and degradation rates are a few aspects that need to be addressed. These flaws can be fixed by optimal design and selection of appropriate fabrication techniques to tailor the ensuing characteristics of these composites for their intended use. Moreover, the reproducibility of the reported research is very challenging, which limits its scalability and commercialization. Nevertheless, the nanofibers-hydrogel-based composites hold a promising future in the biomedical field because of their versatility and superior functionality, which address numerous challenges in targeted drug delivery, tissue engineering, and wound healing applications.

## 4. Concluding Remarks

Composite hydrogel–fiber systems hold immense promise in a range of biomedical applications, including localized cancer therapy, chronic wound healing, bone regeneration, and implantable drug depots. Drug incorporation can occur at multiple stages, either within the fibers or in the hydrogel, or both, allowing for programmable release profiles which can be fine-tuned through material selection, crosslinking density, and environmental responsiveness (e.g., pH- or temperature-sensitive polymers). Their structural versatility, coupled with precise control over drug loading and release, makes them especially attractive for personalized medicine and regenerative therapies.

From a translational perspective, future developments in this field are likely to focus on integrating multifunctional capabilities—for instance, combining drug delivery with biosensing to enable real-time feedback and adaptive dosing. Advances in biofabrication techniques, such as 3D bioprinting and electrospinning–hydrogel hybridization, could further enhance spatial control over drug distribution and mechanical properties.

Emerging strategies that merge 3D printing with electrospinning, particularly through melt electrowriting (MEW), offer a powerful route to generate highly ordered fiber networks within hydrogel matrices. This approach enables the creation of microscale architectures with tailored porosity, anisotropy, and mechanical gradients, which are critical for guiding cell alignment, vascularization, and tissue integration.

Moreover, the convergence of MEW, hydrogel chemistry, and nanofiber functionalization can facilitate the design of hierarchical composites that mimic native extracellular matrices more faithfully, while also supporting on-demand drug release or dynamic responsiveness to physiological cues. Looking ahead, these techniques could allow the fabrication of patient-specific, multi-material constructs that combine structural reinforcement, spatiotemporal control of therapeutics, and bioactive signaling in a single platform.

Despite these advantages, challenges remain in scaling production while maintaining reproducibility, ensuring long-term stability of loaded drugs, and meeting regulatory requirements for combination products. Addressing these issues will require closer collaboration between materials scientists, biomedical engineers, and clinicians to design systems that not only perform optimally in laboratory conditions but also translate effectively into clinical settings.

## Figures and Tables

**Table 1:** Strategies for drug incorporation.

Technique	Advantages	Disadvantages	Key Factors	References
Blending	Simple, straightforward, single step Highest loading rate	Requires a solvent for both the polymer and the drug Burst release is generally observed	Drug/polymer interactions HLB/solubility may compromise homogeneity Diffusion-dependent release profiles	[50,51]
Emulsion electrospinning	Minimizes the contact of the solvent with (sensitive) drugs Standard setup Core–sheath (protective) structures	Additional compositional parameters to optimize (surfactant(s) type(s), concentrations, etc.).	Emulsions must be stable during processing Relative viscosity	[37,52,53,54]
Coaxial electrospinning	Different polymer/drug combinations in core–sheath fibers Modular platform is achievable for multi-drug systems Longer release profiles (when the drug is in the core)	Specific coaxial nozzle required Additional compositional and processing parameters to tune Limited scalability	Processing optimization (core/shell ratio, relative flow rates, etc.) Core–shell thickness allows for fine-tuning of release profiles	[55]
Post-processing surface modification: Physical absorption, coatings, etc.	Simple Avoids contact of the drug with the polymer solvent	Non-covalent (weak) bond Fast release Limited drug incorporation	Polymer-drug interactions	[56]
Post-processing surface modification: Grafting, crosslinking, etc.	Covalent (stronger) bond Readily exposed drugs Avoids contact of the drug with the polymer solvent	Limited drug incorporation Multiple steps	Chemical paths	[57]
Supramolecular-loaded sub-compartments within electrospun fibers	Different protective physical barriers for longer release profiles Suitable for sensitive drugs	Multiple steps	Supramolecular carrier/polymer compatibility	[27]

**Table 3:** Electrospun composite hydrogels for various biomedical applications.

Nanofibers	Hydrogel	Drug/ Cytokine	Technique	Major Findings	Application
Soy protein isolate (SPI)	Methacrylated Chitosan (CS)	Curcumin, Riboflavin	Electrospinning, photo-crosslinking	Anti-inflammatory response of curcumin, fibroblast formation, and epidermal formation due to SPI nanofibers, complete wound closure in 20 days	Epidermal regeneration and wound healing [82]
Poly(oligoethylene glycol methacrylate) (POEGMA)	A-POEGMA and H-POEGMA suspensions	Cellulose nano crystals (CNC)	Electrospinning, spin coating, thermal wrinkling	Enhanced mechanical strength and decreased protein adsorption, tunable nanofiber orientation and density, cell growth, and proliferation	*In vivo* cell screening and *in vivo* tissue regeneration [83]
Polyacrylonitrile (PAN)	Tragacanth gum	Silver Sulfadiazine (SSD), *Aloe vera*	Electrospinning and spraying	Increased swelling, good mechanical properties, 70% SSD release in 72 hrs., cytocompatibility	Wound healing [84]
Poly-(lactic acid-co-trimethylene carbonate) (PLATMC)	Methacrylate Gelatin (GelMa)	Epinecidin-1@chitosan nanoparticles (Epi-1@CS)	Electrospinning, layer stacking, UV irradiation	Cytocompatibility, antioxidant, anti-inflammatory, and antibacterial properties, promoting collagen deposition and angiogenesis	Temperature-responsive composite hydrogel for diabetic-infected wounds [86]
Poly(ester urethane)urea (PEUU)	dECM gel (Porcine dermal tissue)	---	Concurrent electrospinning, electrospraying	Flexible, good mechanical strength, high degree of cellular infiltration	Tissue regeneration [89]
Polylactic acid (PLA)	Hyaluronic acid (HA)	Valsartan, ascorbic acid	Electrospinning, layered deposition	Graded release of VA, increased re-epithelialization, and enhanced collagen deposition	Chronic wound healing [91]
Polyacrylamide6 (PA6)	Grafted PA6	Tallow modified Clay (TMC), Doxycycline hydrochloride drug	Electrospinning, free radical polymerization	Enhanced mechanical strength and swelling ratio, burst release of drug in 2 hrs., antibacterial activity against *E. coli* and *S. aureus*	Drug delivery [100]
Poly-ε-caprolactone (PCL)	F127 hydrogel	Ropivacaine (Rop), Clonidine (Clo)	Electrospinning, mixing	Good *in vivo* biodegradability and biosafety, sustained release of Rop, and sensorimotor segregation effect achieved	Injectable composite for prolonged walking analgesia [101]
Cellulose Acetate (CA)	Poly(acrylamide) (poly-Aam)	Ibuprofen (Ib)	Electrospinning, coating, and photopolymerization	Sustained release of Ib, biocompatibility with 3T3 adipose cells in vitro	Drug delivery system [102]
Poly(L-lactic acid) (PLLA)	Human Elastin-like Polypeptides (HELPs)	---	Electrospinning, deposition	Enhanced wettability, stable HELP moiety	Drug delivery system [103]
Poly-ε-caprolactone (PCL)	Chitosan (CS)	---	Electrospinning, layer-by-layer assembly	Improved porosity and water retention, good mechanical properties, cytocompatibility, cell attachment, proliferation, and infiltration	Skin tissue regeneration and wound healing [104]
Collagen short nanofibers (CSNFs)	Hyaluronic acid (HA), Chondroitin sulfate (CS)	---	Electrospinning, Schiff-base reaction	Cytocompatible, biodegradable, showed chondrogenic differentiation, and no inflammatory response	Tissue engineering and cartilage repair [105]
Silk fibroin/PVA	Sodium Alginate/gum tragacanth (SA/GT)	Cardamom extract	Electrospinning, layered deposition	Good swelling ratio, sustained release of Cardamom extract, biocompatibility, and cell proliferation, antibacterial activity against *E. coli* and *S. aureus*	Wound healing and skin tissue regeneration [106]
Poly-ε-caprolactone (PCL)	EFZ/HG (glycerin) hydrogel matrix	Ritonavir (RIT), Efavirenz (EFZ)	Electrospinning, cryocutting, 3D printing	Good mechanical stability and elongation, in vitro rapid release of EFZ in 45 min, slow RIT release during 72 hrs	Anti-HIV drug delivery system [107]
Methacrylated Gelatin (MGel)	Methacrylated Gelatin (MGel) and Methacrylated Hyaluronic acid (MHA)	Superparamagnetic iron oxide (Fe3O4) nanoparticles as MNPs	Electrospinning, magnetic field-induced nanofiber alignment in hydrogel, in situ crosslinking	Anisotropic morphology, hydrogel promoted myofibroblast differentiation	ECM composition, tissue engineering [108]
Dextran Vinyl Sulfone (DVS)	Dextran Vinyl Sulfone (DVS)	PVP-coated Superparamagnetic iron oxide nanoparticles (SPIONs)	Electrospinning, magnetic field-induced nanofiber alignment in hydrogel, UV-crosslinking	Orthogonal tunability of fiber length, density, alignment, and controlled multidirectional cellular migration	Tissue repair and controlling cell behavior [109]
Core/shell PMMA/silk fibroin nanofibers	Methacrylated gelatin and thiolated pectin	Melatonin (Mel) and Tideglusib (Td)	Coaxial electrospinning	Controlled release of Mel and Td to induce dental pulp stem cell proliferation and odontogenic differentiation	Injectable hydrogels for dental pulp regeneration [110]
Poly(ethylene) oxide (PEO)/Chitosan (CS)	Nanofibrous hydrogel	ZnO-NPs, Pentaerythritol triacrylate (PETA)	Nanospinner electrospinning setup, UV irradiation	High swelling ratio, antibacterial activity against *S. aureus*, *E. coli*, *S. epidermidis*, and *P. aeruginosa*	Antibacterial applications [111]
Poly-ε-caprolactone (PCL)	Poly(ethylene glycol)-poly(e-caprolactone) (PEGPCL)	Nerve growth factor (NGF)	Electrospinning, photo-polymerization	Sustained release of NGF for two weeks, cytocompatibility	Neural prostheses [112]
Collagen or Poly(ε-caprolactone-co-D,L-lactide) (P(CL:DLLA))	Hyaluronan (HA)/Methylcellulose (MC)	Neural stem/progenitor cells (NSPCs)	Electrospinning, dispersion in cell culture	Cytocompatible, in vitro NSPC survival and differentiation	Injectable neural cell distribution and delivery [113]
3D Silk fiber, nylon monofilament, PGLA monofilament	LD- and HD- PEG	---	Thiol-norbornene photoclick Chemistry for hydrogel synthesis, dual-layered stacking	Strong interfacial adhesion, good mechanical strength, controllable degradability, and showed successful chondrogenesis	Articulate cartilage repair [114]
Polycaprolactone (PCL) nanofibrils by hydrolysis	Gelatin and Alginate	Murine fibroblast cell line (NIH3T3)	Electrospinning of PCL nanofibrils	Enhanced mechanical properties, fibroblast showed superior adhesion behavior, and collagen synthesis	Cell cultivation for ECM regeneration [115]
Hyaluronic acid (HA)/Polycaprolactone (PCL)	Gelatin (catechol modified)	Doxorubicin (DOX), cytokines-loaded polyelectrolyte complex nanoparticles (PCNs)	Dual source/dual power electrospinning	Controlled release of DOX and cytokines, inhibition of cancer cell growth	Targeted drug delivery system for osteosarcoma cancer treatment [116]
Poly (D, L-lactic acid) (PDLLA)	Methacrylated Gelatin (GelMA)	---	Core/shell composite produced by coaxial electrospinning	High porosity and water retention promoted endothelial cell proliferation, migration, adhesion, infiltration, and angiogenic differentiation	Diabetic wound healing [117]
PLGA, LA: GA/PCL/Gelatin (PPG)	Polyvinyl alcohol (PVA), collagen	Dopamine	Electrospinning, freeze–thawing	Enhanced mechanical stability during compression, high water absorption and swelling ratio, cell proliferation, adhesion, and growth on porous scaffold	Adipose tissue engineering [118]
Polylactic acid (PLA)	Alginate/Sodium L-lactate	Proteinase K	Electrospinning followed by Plasma treatment	Hydrophilic nanofibers, burst release of lactate followed by sustained release for ten days	Controlled drug release [119]
Polydopamine (PDA)/Polyethylene oxide (PEO), Zein	Gelatin, Polyethylene imine (PEI), PDA, Zein	Tetracycline hydrochloride (T)	Core–shell nanofibers by coaxial electrospinning	Water retention, swell ability, burst release of T, antibacterial resistance against *E. coli* and *S. aureus*	Wound healing [120]
Ethyl cellulose (EC) nanofibers	Carboxymethyl cellulose (CMC) film	Phenytoin (PHT), Tetracycline hydrochloride (TCH)	Electrospinning, solvent casting, fiber-on-film, and fiber-in-film	Fiber-in-film composite showed stage release of TCH and PHT in 8 hrs., fiber-on-film composite showed simultaneous release of TCH and PHT	Modulated drug delivery system [79]
Polycaprolactone (PCL)	Sodium Alginate-Gelatin	Amoxicillin (AMX), Epidermal growth factor (rhEGF)	Electrospinning, 3D printing	Showed good mechanical properties, both hydrophobic outer and hydrophilic inner, good cell adhesion, and proliferation	Wound healing applications [121]
Poly(lactic acid) (PLA)	Alginate-graft-hyaluronate (Alg-g-HA)	Chondrocytes	Electrospinning, hydrogel with nanofiber suspension	Higher compressive modulus, cytocompatible, produced cartilage matrix	Cartilage tissue regeneration [122]
Polycaprolactone/Gelatin	Alginate sulfate	Human adipose-derived stem cells (hASCs), powdered ECM	Electrospinning	Enhanced cell proliferation and chondrogenic differentiation	Cartilage tissue engineering [123]
Poly(L-lactide) (PLLA)	P(NIPAAm-co-NIPMAAm)	Rhodamine B, Gold nanorods (AuNRs)	Electrospinning, UV irradiation for crosslinking	NIR thermoresponse of hydrogel, sustained drug release, and penetration	Thermoresponsive hydrogel for controlled drug delivery [124]
Poly-(γ-benzyl-L-glutamate) (PBLG) Poly(l-lactide-co-ε-caprolactone) (PLCL)/gelatin methacryloyl (GelMA)/alginate	PRONOVA SLG100 Alginate (NovaMatrix, Norway)	Vascular endothelial growth factor (VEGF)	Core/shell coaxial electrospinning	Enhanced mechanical strength, great cell viability, and VEGF release in two weeks	Angiogenic factor delivery for beta cell therapy to treat diabetes mellitus [125]
Poly(lactic-co-glycolic acid) (PLGA) ANFs	Collagen and GelMA-PEO	Cardiac fibroblasts (CFs), HL-1 Cardiomyocytes	Aligned electrospinning	Highly oriented nanofibers, uniform length and diameter, high cell viability, aligned tissue growth	Anisotropic engineered tissue [126]
Polylactic acid (PLA), PLA-b- PDMAEMA	Carboxy-methylcellulose (CMC)	---	Spin coating and electrospinning, UV crosslinking	Increase in storage modulus, good reinforcement effect, improved hydrophilicity	Injectable composite systems for biomedical applications [127]
Polyhydroxy butyrate (PHB)	Methacrylated Gelatin	Bioactive HAp nanoparticles (bone mineral)	Electrospinning, UV crosslinking	Good mechanical properties, bone cell viability, and infiltration for 14 days	Bone tissue regeneration [128]

## Data Availability

No new data were created or analyzed in this study.

## References

[1] Zhang M., Xu S., Wang R., Che Y., Han C., Feng W., Wang C., Zhao W. (2023). Electrospun nanofiber/hydrogel composite materials and their tissue engineering applications. J. Mater. Sci. Technol..

[2] Patel D.K., Won S.-Y., Jung E., Han S.S. (2025). Recent progress in biopolymer-based electrospun nanofibers and their potential biomedical applications: A review. Int. J. Biol. Macromol..

[3] Shabani A., Al G.A., Berri N., Castro-Dominguez B., Leese H.S., Martinez-Hernandez U. (2025). Electrospinning technology, machine learning, and control approaches: A review. Adv. Eng. Mater..

[4] Choi C., Yun E., Cha C. (2023). Emerging technology of nanofiber-composite hydrogels for biomedical applications. Macromol. Biosci..

[5] Nanda D., Behera D., Pattnaik S.S., Sahoo D., Panigrahi S.K., Mallick B.C. (2025). Advances in natural polymer-based hydrogels: Synthesis, applications, and future directions in biomedical and environmental fields. Discov. Polym..

[6] Liu Z., Ma X., Liu J., Zhang H., Fu D. (2025). Advances in the application of natural/synthetic hybrid hydrogels in tissue engineering and delivery systems: A comprehensive review. Int. J. Pharm..

[7] Khan M.U.A., Aslam M.A., Abdullah M.F.B., Al-Arjan W.S., Stojanovic G.M., Hasan A. (2024). Hydrogels: Classifications, fundamental properties, applications, and scopes in recent advances in tissue engineering and regenerative medicine—A comprehensive review. Arab. J. Chem..

[8] Protsak I.S., Morozov Y.M. (2025). Fundamentals and advances in stimuli-responsive hydrogels and their applications: A review. Gels.

[9] Santhamoorthy M., Kim S.-C. (2025). A review of the development of biopolymer hydrogel-based scaffold materials for drug delivery and tissue engineering applications. Gels.

[10] Ghosh T., Das T., Purwar R. (2021). Review of electrospun hydrogel nanofiber system: Synthesis, properties and applications. Polym. Eng. Sci..

[11] Sheffield C., Meyers K., Johnson E., Rajachar R.M. (2018). Application of composite hydrogels to control physical properties in tissue engineering and regenerative medicine. Gels.

[12] Mele E., Kny E., Ghosal K., Thomas S. (2018). Chapter 3: Biomimetic electrospun composites: From fundamental insights to commercialization. Electrospinning: From Basic Research to Commercialization.

[13] Thenmozhi S., Dharmaraj N., Kadirvelu K., Kim H.Y. (2017). Electrospun nanofibers: New generation materials for advanced applications. Mater. Sci. Eng. B.

[14] Kong B., Liu R., Guo J., Lu L., Zhou Q., Zhao Y. (2023). Tailoring micro/nano-fibers for biomedical applications. Bioact. Mater..

[15] Ahadian S., Obregón R., Ramón-Azcón J., Salazar G., Ramalingam M., Ramalingam M., Ramakrishna S. (2017). Clinical/preclinical aspects of nanofiber composites. Nanofiber Composites for Biomedical Applications.

[16] Murugupandian R., Suresh A.S., Vijaylal L., John A.E., Uthirapathy V. (2022). A review on nanofibrous scaffolding technique for potential tissue engineering applications. Trends Biomater. Artif. Organs.

[17] Rodríguez-Tobías H., Morales G., Grande D. (2019). Comprehensive review on electrospinning techniques as versatile approaches toward antimicrobial biopolymeric composite fibers. Mater. Sci. Eng. C.

[18] Aldana A.A., Abraham G.A. (2017). Current advances in electrospun gelatin-based scaffolds for tissue engineering applications. Int. J. Pharm..

[19] Popov Pereira da Cunha M.D., Caracciolo P.C., Abraham G.A. (2021). Latest advances in electrospun plant-derived protein scaffolds for biomedical applications. Curr. Opin. Biomed. Eng..

[20] Montini-Ballarin F., Calvo D., Caracciolo P.C., Rojo F., Frontini P.M., Abraham G.A., Guinea G.V. (2016). Mechanical behavior of bilayered small-diameter nanofibrous structures as biomimetic vascular grafts. J. Mech. Behav. Biomed. Mater..

[21] Sridhar R., Lakshminarayanan R., Madhaiyan K., Amutha Barathi V., Lim K.H.C., Ramakrishna S. (2015). Electrosprayed nanoparticles and electrospun nanofibers based on natural materials: Applications in tissue regeneration, drug delivery and pharmaceuticals. Chem. Soc. Rev..

[22] Chen K., Hu H., Zeng Y., Pan H., Wang S., Zhang Y., Shi L., Tan G., Pan W., Liu H. (2022). Recent advances in electrospun nanofibers for wound dressing. Eur. Polym. J..

[23] Ghosal K., Agatemor C., Špitálsky Z., Thomas S., Kny E. (2019). Electrospinning tissue engineering and wound dressing scaffolds from polymer–titanium dioxide nanocomposites. Chem. Eng. J..

[24] Rimoli I.H., Unalan I., Mutlu N., Michálek M., Abraham G.A., Liverani L., Boccaccini A.R. (2024). Cotton wool-like ion-doped bioactive glass nanofibers: Investigation of Zn and Cu combined effect. Biomed. Mater..

[25] Kajdič S., Planinšek O., Gašperlin M., Kocbek P. (2019). Electrospun nanofibers for customized drug-delivery systems. J. Drug Deliv. Sci. Technol..

[26] Thakkar S., Misra M. (2017). Electrospun polymeric nanofibers: New horizons in drug delivery. Eur. J. Pharm. Sci..

[27] Sonzogni A., Rivero G., González V., Abraham G.A., Calderón M., Minari R. (2024). Nano-in-nano enteric protein delivery system: Coaxial Eudragit® L100-55 fibers containing poly(N-vinylcaprolactam) nanogels. Biomater. Sci..

[28] Majumder S., Sagor M.M.H., Arafat M.T. (2022). Functional electrospun polymeric materials for bioelectronic devices: A review. Mater. Adv..

[29] Halicka K., Cabaj J. (2021). Electrospun nanofibers for sensing and biosensing applications—A review. Int. J. Mol. Sci..

[30] Silva J.A., De Gregorio P.R., Rivero G., Abraham G.A., Nader-Macías M.E.F. (2021). Immobilization of vaginal *Lactobacillus* in polymeric nanofibers for its incorporation in vaginal probiotic products. Eur. J. Pharm. Sci..

[31] Rather A.H., Khan R.S., Wani T.U., Beigh M.A., Sheikh F.A. (2022). Overview on immobilization of enzymes on synthetic polymeric nanofibers fabricated by electrospinning. Biotechnol. Bioeng..

[32] Liu Z., Ramakrishna S., Liu X. (2020). Electrospinning and emerging healthcare and medicine possibilities. APL Bioeng..

[33] Reddy V.S., Tian Y., Zhang C., Ye Z., Roy K., Chinnappan A., Ramakrishna S., Liu W., Ghosh R. (2021). A review on electrospun nanofibers based advanced applications: From health care to energy devices. Polymers.

[34] Sánchez Cerviño M.C., Fuioaga C.P., Atanase L.I., Abraham G.A., Rivero G. (2023). Electrohydrodynamic techniques for the manufacture and/or immobilization of vesicles. Polymers.

[35] Ding J., Zhang J., Li J., Li D., Xiao C., Xiao H., Yang H., Zhuang X., Chen X. (2019). Electrospun polymer biomaterials. Prog. Polym. Sci..

[36] Wang C., Wang J., Zeng L., Qiao Z., Liu X., Liu H., Zhang J., Ding J. (2019). Fabrication of electrospun polymer nanofibers with diverse morphologies. Molecules.

[37] Yoo H.S., Kim T.G., Park T.G. (2009). Surface-functionalized electrospun nanofibers for tissue engineering and drug delivery. Adv. Drug Deliv. Rev..

[38] Bongiovanni Abel S., Montini Ballarin F., Abraham G.A. (2020). Combination of electrospinning with other techniques for the fabrication of 3D polymeric and composite nanofibrous scaffolds with improved cellular interactions. Nanotechnology.

[39] Persano L., Camposeo A., Tekmen C., Pisignano D. (2013). Industrial upscaling of electrospinning and applications of polymer nanofibers: A review. Macromol. Mater. Eng..

[40] Kim I.G., Lee J.-H., Unnithan A.R., Park C.-H., Kim C.S. (2015). A comprehensive electric field analysis of cylinder-type multi-nozzle electrospinning system for mass production of nanofibers. J. Ind. Eng. Chem..

[41] Sill T.J., von Recum H.A. (2008). Electrospinning: Applications in drug delivery and tissue engineering. Biomaterials.

[42] Bock N., Dargaville T.R., Woodruff M.A. (2012). Electrospraying of polymers with therapeutic molecules: State of the art. Top. Issue Polym. Biomater..

[43] Greiner A., Wendorff J.H. (2008). Functional self-assembled nanofibers by electrospinning. Adv. Polym. Sci..

[44] Avossa J., Herwig G., Toncelli C., Itel F., Rossi R.M. (2022). Electrospinning based on benign solvents: Current definitions, implications and strategies. Green Chem..

[45] Bhardwaj N., Kundu S.C. (2010). Electrospinning: A fascinating fiber fabrication technique. Biotechnol. Adv..

[46] De Vrieze S., Van Camp T., Nelvig A., Hagström B., Westbroek P., De Clerck K. (2009). The effect of temperature and humidity on electrospinning. J. Mater. Sci..

[47] Mailley D., Hébraud A., Schlatter G. (2021). A review on the impact of humidity during electrospinning: From the nanofiber structure engineering to the applications. Macromol. Mater. Eng..

[48] Williams G.R., Raimi-Abraham B.T., Luo C.J. (2018). Monoaxial electrospinning. Nanofibres in Drug Delivery.

[49] Kenry, Lim C.T. (2017). Nanofiber technology: Current status and emerging developments. Prog. Polym. Sci..

[50] Luraghi A., Peri F., Moroni L. (2021). Electrospinning for drug delivery applications: A review. J. Control. Release.

[51] Cornejo Bravo J.M., Villarreal Gómez L.J., Serrano Medina A., Haider S., Haider A. (2016). Electrospinning for drug delivery systems: Drug incorporation techniques. Electrospinning—Material, Techniques, and Biomedical Applications.

[52] Giannetti R., Abraham G.A., Rivero G. (2019). The role of emulsion parameters in tramadol sustained-release from electrospun mats. Mater. Sci. Eng. C.

[53] Rivero G., Aldana A.A., Frontini Lopez Y.R., Liverani L., Boccaccini A.R., Bustos D.M., Abraham G.A. (2019). 14-3-3ε protein-immobilized PCL-HA electrospun scaffolds with enhanced osteogenicity. J. Mater. Sci. Mater. Med..

[54] Chou S.-F., Carson D., Woodrow K.A. (2015). Current strategies for sustaining drug release from electrospun nanofibers. J. Control. Release.

[55] Pant B., Park M., Park S.-J. (2019). Drug delivery applications of core-sheath nanofibers prepared by coaxial electrospinning: A review. Pharmaceutics.

[56] Meinel A., Germershaus O., Luhmann T., Merkle H., Meinel L. (2012). Electrospun matrices for localized drug delivery: Current technologies and selected biomedical applications. Eur. J. Pharm. Biopharm..

[57] Shabanloo R., Montazer M., Farahani A., Karimi N. (2025). A review on surface modification of nanofibrous textiles for diverse applications: Focus on medical uses. Heliyon.

[58] Elfawal G.F., Šišková A.O., Andicsová A.E. (2025). Electrospinning: A game-changer in fiber production and practical applications. Fibers Polym..

[59] Ejiohuo O. (2023). A perspective on the synergistic use of 3D printing and electrospinning to improve nanomaterials for biomedical applications. Nano Trends.

[60] Yeo M., Kim G. (2020). Micro/nano-hierarchical scaffold fabricated using a cell electrospinning/3D printing process for co-culturing myoblasts and HUVECs to induce myoblast alignment and differentiation. Acta Biomater..

[61] Dalton P.D., Woodfield T.B.F., Mironov V., Groll J. (2020). Advances in hybrid fabrication toward hierarchical tissue constructs. Adv. Sci..

[62] Khrystonko O., Rimpelová S., Burianová T., Švorčík V., Lyutakov O., Elashnikov R. (2023). Smart multi stimuli-responsive electrospun nanofibers for on-demand drug release. J. Colloid Interface Sci..

[63] Tayebi-Khorrami V., Rahmanian-Devin P., Reza Fadaei M., Movaffagh J., Reza Askari V. (2024). Advanced applications of smart electrospun nanofibers in biomedical fields. Mater. Sci. Eng. R Rep..

[64] Vannaladsaysy V., Choudhury S., Datta S., Chatterjee K. (2025). NIR-responsive shape memory composite nanofibers as deployable matrices for biomedical applications. Smart Mater. Struct..

[65] Ferraris S., Spriano S., Calogero Scalia A., Cochis A., Rimondini L., Cruz-Maya I., Guarino V., Varesano A., Vineis C. (2020). Topographical and biomechanical guidance of electrospun fibers for biomedical applications. Polymers.

[66] Gürtler A., Lang J., Czyrski G., Sirois J., Melican K., Rades T., Heinz A. (2025). Electrospun fiber patches for inflammatory skin diseases—Correlating in vitro drug release with ex vivo permeation. Biomater. Adv..

[67] Záhonyi P., Müncz A.G., Péter-Haraszti A., Nagy Z., Csontos I., Marosi G., Szabó E. (2025). Continuous twin-screw melt granulation of drug-loaded electrospun fibers. Eur. J. Pharm. Biopharm..

[68] Yi L., Shi L., Móczó J., Pukánszky B. (2024). Encapsulation of a drug into electrospun fibers spun from water soluble polymers to control solubility and release. Heliyon.

[69] Joy N., Venugopal D., Gopinath A., Samavedi S. (2024). Connecting in situ cone/jet length in electrospinning to fiber diameter and drug release for the rational design of electrospun drug carriers. Chem. Eng. Sci..

[70] Carvalho G., Coimbra P. (2023). Electrospun composite fibers of poly(lactic acid) and halloysite for the sustained co-delivery of drugs with opposite water affinities. Appl. Clay Sci..

[71] Geng Y., Williams G. (2023). Developing and scaling up captopril-loaded electrospun ethyl cellulose fibers for sustained-release floating drug delivery. Int. J. Pharm..

[72] Rajabi T., Naffakh-Moosavy H., Bagheri F. (2024). The synergic effect of nanosecond fiber laser and drug-loaded electrospun PVA coating on metallurgical and biological characteristics of Ti-6Al-4V alloy. Appl. Surf. Sci. Adv..

[73] Azizoğlu G., Azizoğlu E., Barker T., Özer O. (2023). Single and multi-dose drug loaded electrospun fiber mats for wound healing applications. J. Drug Deliv. Sci. Technol..

[74] Mirek A., Grzeczkowicz M., Belaid H., Bartkowiak A., Barranger F., Abid M., Wasyłeczko M., Pogorielov M., Bechelany M., Lewińska D. (2023). Electrospun UV-cross-linked polyvinylpyrrolidone fibers modified with polycaprolactone/polyethersulfone microspheres for drug delivery. Biomater. Adv..

[75] Wang M., Ge R., Zhang F., Yu D., Liu Z., Li X., Shen H., Williams G. (2023). Electrospun fibers with blank surface and inner drug gradient for improving sustained release. Biomater. Adv..

[76] Yi L., Hegyesi N., Móczó J., Pukánszky B. (2024). Improved release of sulfamethoxazole from electrospun water soluble fibers. Polymer.

[77] Gürtler A., Maltschik A., Güler Yildiz S., Vangelofski K., Gade L., Grohganz H., Rades T., Heinz A. (2024). Advancing inflammatory skin disease therapy: Sustained tofacitinib release via electrospun fiber dressings. Eur. J. Pharm. Biopharm..

[78] Fazekas B., Péterfi O., Galata D., Nagy Z., Hirsch E. (2024). Process analytical technology based quality assurance of API concentration and fiber diameter of electrospun amorphous solid dispersions. Eur. J. Pharm. Biopharm..

[79] Mohamed R., Chou S. (2024). Physicomechanical characterizations and in vitro release studies of electrospun ethyl cellulose fibers, solvent cast carboxymethyl cellulose films, and their composites. Int. J. Biol. Macromol..

[80] Cobarrubias-Carapia S., Cervantes-Chávez J., Rojas-Avelizapa N., Luna-Barcenas G., Amaro-Reyes A., Pool H., Villaseñor-Ortega F. (2025). Development of an antioxidant and anti-lipid peroxidation film based on quercetin-loaded Eudragit EPO®/sodium hyaluronate electrospun fibers. Mater. Lett..

[81] Musciacchio L., Mardirossian M., Marussi G., Crosera M., Turco G., Porrelli D. (2025). Core-shell electrospun polycaprolactone nanofibers, loaded with rifampicin and coated with silver nanoparticles, for tissue engineering applications. Biomater. Adv..

[82] Elyasifar N., Samani S., Beheshtizadeh N., Farzin A., Samadikuchaksaraei A., Ai J., Ebrahimi-Barough S., Milan P.B., Haramshahi S.M.A., Azami M. (2023). Bi-layered photocrosslinkable chitosan-curcumin hydrogel/soy protein nanofibrous mat skin substitute. Materialia.

[83] De France K.J., Xu F., Toufanian S., Chan K.J., Said S., Stimpson T.C., González-Martínez E., Moran-Mirabal J.M., Cranston E.D., Hoare T. (2021). Multi-scale structuring of cell-instructive cellulose nanocrystal composite hydrogel sheets via sequential electrospinning and thermal wrinkling. Acta Biomater..

[84] Alvandi H., Jaymand M., Eskandari M., Aghaz F., Hosseinzadeh L., Heydari M., Arkan E. (2023). A sandwich electrospun nanofibers/Tragacanth hydrogel composite containing Aloe vera extract and silver sulfadiazine as a wound dressing. Polym. Bull..

[85] Niemczyk-Soczynska B., Zaszczyńska A., Zabielski K., Sajkiewicz P. (2021). Hydrogel, electrospun and composite materials for bone/cartilage and neural tissue engineering. Materials.

[86] Huang Y., Song M., Li X., Du Y., Gao Z., Zhao Y.-Q., Li C., Yan H., Mo X., Wang C. (2024). Temperature-responsive self-contraction nanofiber/hydrogel composite dressing facilitates the healing of diabetic-infected wounds. Mater. Today Bio.

[87] Wu B., Yang J., Zu Y., Chi J., Shi K. (2022). Aligned electrospun fiber film loaded with multi-enzyme mimetic iridium nanozymes for wound healing. J. Nanobiotechnol..

[88] Liguori A., Gino M., Panzavolta S., Torricelli P., Maglio M., Parrilli A., Gualandi C., Griffoni C., Brodano G.B., Fini M. (2022). Tantalum nanoparticles enhance the osteoinductivity of multiscale composites based on poly(lactide-co-glycolide) electrospun fibers embedded in a gelatin hydrogel. Mater. Today Chem..

[89] Hong Y., Huber A., Takanari K., Amoroso N.J., Hashizume R., Badylak S.F., Wagner W.R. (2011). Mechanical properties and in vivo behavior of a biodegradable synthetic polymer microfiber-extracellular matrix hydrogel biohybrid scaffold. Biomaterials.

[90] Zhao X., Ding M., Xu C., Zhang X., Liu S., Lin X., Wang L., Xia Y. (2021). A self-reinforcing strategy enables the intimate interface for anisotropic alginate composite hydrogels. Carbohydr. Polym..

[91] Ilomuanya M.O., Okafor P.S., Amajuoyi J.N., Onyejekwe J.C., Okubanjo O.O., Adeosun S.O., Silva B.O. (2020). Polylactic acid-based electrospun fiber and hyaluronic acid-valsartan hydrogel scaffold for chronic wound healing. Beni Suef Univ. J. Basic Appl. Sci..

[92] Riaz T., Gull N., Islam A., Dilshad M.R., Atanase L.I., Delaite C. (2023). Needleless electrospinning of poly(ε-caprolactone) nanofibers deposited on gelatin film for controlled release of Ibuprofen. Chem. Pap..

[93] Zare P., Pezeshki-Modaress M., Davachi S.M., Zare P., Yazdian F., Simorgh S., Ghanbari H., Rashedi H., Bagher Z. (2021). Alginate sulfate-based hydrogel/nanofiber composite scaffold with controlled Kartogenin delivery for tissue engineering. Carbohydr. Polym..

[94] Riaz T., Khenoussi N., Rata D.M., Atanase L.I., Adolphe D.C., Delaite C. (2023). Blend electrospinning of poly(ϵ-caprolactone) and poly(ethylene glycol-400) nanofibers loaded with ibuprofen as a potential drug delivery system for wound dressings. Autex Res. J..

[95] Rehman N., Dilshad M.R., Islam A., Gull N., Riaz T., Khan S.M., Khan R.U. (2021). Novel graphene oxide loaded sodium alginate hydrogels cross-linked with tetraethyl orthosilicate for cephradine release analysis. J. Drug Deliv. Sci. Technol..

[96] Ghauri Z.H., Islam A., Qadir M.A., Gull N., Haider B., Khan R.U., Riaz T. (2021). Development and evaluation of pH-sensitive biodegradable ternary blended hydrogel films (chitosan/guar gum/PVP) for drug delivery application. Sci. Rep..

[97] Qiu Y., Park K. (2001). Environment-sensitive hydrogels for drug delivery. Adv. Drug Deliv. Rev..

[98] Xu S., Deng L., Zhang J., Yin L., Dong A. (2016). Composites of electrospun-fibers and hydrogels: A potential solution to current challenges in biological and biomedical field. J. Biomed. Mater. Res. B Appl. Biomater..

[99] Zhong H., Huang J., Luo M., Fang Y., Zeng X., Wu J., Du J. (2023). Near-field electrospun PCL fibers/GelMA hydrogel composite dressing with controlled deferoxamine-release ability and retiform surface for diabetic wound healing. Nano Res..

[100] Shehab-ElDin A.N., Sobh R.A., Rabie A.M., Mohamed W.S., Nasr H.E. (2022). Polyacrylamide grafted electrospun polyamide 6 nanocomposite fibers for drug delivery application. Egypt J. Chem..

[101] Chen S., Yao W., Wang H., Wang T., Xiao X., Sun G., Yang J., Guan Y., Zhang Z., Xia Z. (2022). Injectable electrospun fiber-hydrogel composite sequentially releasing clonidine and ropivacaine for prolonged and walking regional analgesia. Theranostics.

[102] Attia M.F., Montaser A.S., Arifuzzaman M., Pitz M., Jlassi K., Alexander-Bryant A., Kelly S.S., Alexis F., Whitehead D.C. (2021). In situ photopolymerization of acrylamide hydrogel to coat cellulose acetate nanofibers for drug delivery system. Polymers.

[103] Bandiera A., Passamonti S., Dolci L.S., Focarete M.L. (2018). Composite of elastin-based matrix and electrospun poly(L-lactic acid) fibers: A potential smart drug delivery system. Front. Bioeng. Biotechnol..

[104] Irantash S., Gholipour-Kanani A., Najmoddin N., Varsei M. (2024). A hybrid structure based on silk fibroin/PVA nanofibers and alginate/gum tragacanth hydrogel embedded with cardamom extract. Sci. Rep..

[105] Darwesh A.Y., Helmy A.M., Abdelhakk H.M., Giri B., Maniruzzaman M. (2024). 3D-printed short nanofibers/hydrogel-based vaginal films as a novel system for the delivery of anti-HIV microbicide drugs. J. Drug Deliv. Sci. Technol..

[106] Teixeira M.O., Antunes J.C., Felgueiras H.P. (2021). Recent advances in fiber–hydrogel composites for wound healing and drug delivery systems. Antibiotics.

[107] McMurtrey R.J. (2014). Patterned and functionalized nanofiber scaffolds in three-dimensional hydrogel constructs enhance neurite outgrowth and directional control. J. Neural Eng..

[108] Choi C., Yun E., Song M., Kim J., Son J.S., Cha C. (2024). Multiscale control of nanofiber-composite hydrogel for complex 3D cell culture by extracellular matrix composition and nanofiber alignment. Biomater. Res..

[109] Hiraki H.L., Matera D.L., Rose M.J., Kent R.N., Todd C.W., Stout M.E., Wank A.E., Schiavone M.C., DePalma S.J., Zarouk A.A. (2021). Magnetic alignment of electrospun fiber segments within a hydrogel composite guides cell spreading and migration phenotype switching. Front. Bioeng. Biotechnol..

[110] Atila D., Keskin D., Lee Y.L., Lin F.H., Hasirci V., Tezcaner A. (2023). Injectable methacrylated gelatin/thiolated pectin hydrogels carrying melatonin/tideglusib-loaded core/shell PMMA/silk fibroin electrospun fibers for vital pulp regeneration. Colloids Surf. B Biointerfaces.

[111] Estrada-Villegas G.M., Del Rio-De Vicente J.I., Argueta-Figueroa L., Gonzalez-Perez G. (2020). UV-initiated crosslinking of electrospun chitosan/poly(ethylene oxide) nanofibers doped with ZnO-nanoparticles: Development of antibacterial nanofibrous hydrogel. MRS Commun..

[112] Han N., Rao S.S., Johnson J., Parikh K.S., Bradley P.A., Lannutti J.J., Winter J.O. (2011). Hydrogel-electrospun fiber mat composite coatings for neural prostheses. Front. Neuroeng..

[113] Hsieh A., Zahir T., Lapitsky Y., Amsden B., Wan W., Shoichet M.S. (2010). Hydrogel/electrospun fiber composites influence neural stem/progenitor cell fate. Soft Matter.

[114] Kim J.S., Choi J., Ki C.S., Lee K.H. (2021). 3D silk fiber construct embedded dual-layer PEG hydrogel for articular cartilage repair—In vitro assessment. Front. Bioeng. Biotechnol..

[115] Lee S., Kim H.S., Yoo H.S. (2017). Electrospun nanofibrils embedded hydrogel composites for cell cultivation in a biomimetic environment. RSC Adv..

[116] Lee C.-H., Huang W.-Y., Lee K.-Y., Kuan C.-H., Wu T.-C., Sun J.-S., Wang T.-W. (2024). Bioinspired adhesive nanofibrous hydrogel promotes immune infiltration through effective immunochemotherapy for osteosarcoma treatment. Chem. Eng. J..

[117] Li J., Zhang T., Pan M., Xue F., Lv F., Ke Q., Xu H. (2022). Nanofiber/hydrogel core–shell scaffolds with three-dimensional multilayer patterned structure for accelerating diabetic wound healing. J. Nanobiotechnol..

[118] Li C., Ge J., Guo Q., Wang J., Wu J., Yan Z., Špitalský Z., Liu Y. (2024). Polyvinyl alcohol/collagen composite scaffold reinforced with biodegradable polyesters/gelatin nanofibers for adipose tissue engineering. Int. J. Biol. Macromol..

[119] Macor L.P., Colombi S., Tamarit J.-L., Engel E., Pérez-Madrigal M.M., García-Torres J., Alemán C. (2023). Immediate-sustained lactate release using alginate hydrogel assembled to proteinase K/polymer electrospun fibers. Int. J. Biol. Macromol..

[120] Martin A., Nyman J.N., Reinholdt R., Cai J., Schaedel A.L., van der Plas M.J.A., Malmsten M., Rades T., Heinz A. (2022). In situ transformation of electrospun nanofibers into nanofiber-reinforced hydrogels. Nanomaterials.

[121] Song Y., Hu Q., Liu S., Wang Y., Zhang H., Chen J., Yao G. (2024). Electrospinning/3D printing drug-loaded antibacterial polycaprolactone nanofiber/sodium alginate-gelatin hydrogel bilayer scaffold for skin wound repair. Int. J. Biol. Macromol..

[122] Mohabatpour F., Karkhaneh A., Sharifi A.M. (2016). A hydrogel/fiber composite scaffold for chondrocyte encapsulation in cartilage tissue regeneration. RSC Adv..

[123] Najafi R., Chahsetareh H., Pezeshki-Modaress M., Aleemardani M., Simorgh S., Davachi S.M., Alizadeh R., Asghari A., Hassanzadeh S., Bagher Z. (2023). Alginate sulfate/ECM composite hydrogel containing electrospun nanofiber with encapsulated human adipose-derived stem cells for cartilage tissue engineering. Int. J. Biol. Macromol..

[124] Nakielski P., Pawłowska S., Rinoldi C., Ziai Y., De Sio L., Urbanek O., Zembrzycki K., Pruchniewski M., Lanzi M., Salatelli E. (2020). Multifunctional platform based on electrospun nanofibers and plasmonic hydrogel: A smart nanostructured pillow for near-infrared light-driven biomedical applications. ACS Appl. Mater. Interfaces.

[125] Toftdal M.S., Christensen N.P., Kadumudi F.B., Dolatshahi-Pirouz A., Grunnet L.G., Chen M. (2024). Mechanically reinforced hydrogel vehicle delivering angiogenic factor for beta cell therapy. J. Colloid Interface Sci..

[126] Zhang J.Y., Cheraga N., Huang N.P. (2022). 3D cell/scaffold model based on aligned-electrospun-nanofiber film/hydrogel multi-layers for construction of anisotropic engineered tissue. Biointerphases.

[127] Zhang X., Megone W., Peijs T., Gautrot J.E. (2020). Functionalization of electrospun PLA fibers using amphiphilic block copolymers for use in carboxymethyl-cellulose hydrogel composites. Nanocomposites.

[128] Sadat-Shojai M., Khorasani M.T., Jamshidi A. (2016). A new strategy for fabrication of bone scaffolds using electrospun nano-HAp/PHB fibers and protein hydrogels. Chem. Eng. J..

[129] Barzegar A., Ebrahimzadeh S., Vahdani V., Tohidifar N., Zarrini G., Hatami H., Nikzad B., Warda M., Hacimuftuoglu A. (2024). Engineering bi-layered skin-like nanopads with electrospun nanofibers and chitosan films for promoting fibroblast infiltration in tissue regeneration and wound healing. Int. J. Biol. Macromol..

[130] Karimizade A., Hasanzadeh E., Abasi M., Enderami S.E., Mirzaei E., Annabi N., Mellati A. (2024). Collagen short nanofiber-embedded chondroitin sulfate–hyaluronic acid nanocomposite: A cartilage-mimicking in situ-forming hydrogel with fine-tuned properties. Int. J. Biol. Macromol..

[131] Rată D.M., Cadinoiu A.N., Vochița G., Gherghel D., Lakkaboyana S.K., Fuioagă C.P. (2025). Biocomposite complex hydrogels with antimicrobial activity suitable for wound healing. J. Polym. Sci..

